# A Questionnaire Survey of Prescription Preferences and Leftover Medication Conversations: Comparisons Among Kidney Disease Patients and Healthcare Professionals

**DOI:** 10.7759/cureus.45842

**Published:** 2023-09-24

**Authors:** Takeshi Nakata, Akihiro Fukuda, Kyoko Ojiro, Kazuhiro Matsuyama, Takayuki Masaki, Hiroki Itoh, Hirotaka Shibata

**Affiliations:** 1 Department of Endocrinology Metabolism, Rheumatology, and Nephrology, Faculty of Medicine, Oita University, Oita, JPN; 2 Department of Internal Medicine, Matsuoka Medical Clinic, Oita, JPN; 3 Department of Nephrology, Matsuyama Clinic, Oita, JPN; 4 Department of Clinical Pharmacy, Oita University Hospital, Oita, JPN

**Keywords:** leftover medication, hemodialysis, compliance, adherence, questionnaire-based survey

## Abstract

Background: Patients with chronic kidney disease (CKD) and patients with kidney failure receiving hemodialysis (HD) receive various types of medications. However, little is known about the differences in medication preference and how to deal with leftover medication among CKD patients and HD patients. The purpose of this study was to investigate the differences in medication preference and ways of dealing with leftover medication between CKD patients, HD patients, physicians, and pharmacists via a questionnaire survey.

Methods: The ethics committee of Oita University, Oita, Japan, approved this survey. Outpatients undergoing treatment by a nephrologist in four facilities in Oita prefecture, Japan, were asked to answer a questionnaire on their preference for medication and how to deal with leftover medication. Respondents gave their informed written consent. The same questionnaire was administered to nephrologists and pharmacists online.

Results: In this survey, 383 patients (260 patients with CKD and 123 patients with HD), 22 nephrologists, and 28 pharmacists responded. The response rate of valid responses was more than 90% for each of the groups. In particular, 41% of patients with CKD and 56% of patients with HD never inform their doctor about leftover medication or only inform them when there is a lot of leftover medication. On the other hand, 23% of physicians have never asked their patients about them. Ordinary logistic regression analysis indicated that there is no significant relationship between how often patients talk about leftover medication, patients’ preferences, or patient states.

Conclusions: Despite the age and state of the patients, it is important to discuss the perception of medication with each other and confirm the condition of the remaining medication to improve concordance and obtain the desired treatment effect.

## Introduction

Adherence of patients plays an important role, especially in chronic diseases such as hypertension and chronic kidney disease (CKD). Although measuring adherence is difficult, the World Health Organization (WHO) has estimated adherence to be only about 50% for chronic diseases [1]. Poor adherence might lead to a poor prognosis and require an additional medical fee from patients and governments [2,3]. Chronic kidney disease patients and patients with hemodialysis (HD) have also been reported to have low adherence [3].

The frequency, pill counts, and forms of medication could affect adherence. Recently, new types of medications have been developed, such as long-acting medications (once a week) or chewable medications that can be taken without water. There are many types of phosphate binders, including tablets, chewable tablets, and powders, but people with CKD and HD face a number of problems when using phosphate binders on a daily basis. [4,5].

Little is known about the drug preference for medications, especially in terms of the differences between CKD patients, HD patients, and healthcare professionals. Ordinary CKD patients go to the hospital only once a month or once every two or three months. On the other hand, HD patients go to the hospital three times a week. These differences may affect the patient's awareness or acceptance of their illness. Acceptance of your own illness is an important factor in adherence [6]. Furthermore, ordinary HD patients must take more medications than CKD patients [7,8]. We see from previous research that the perceived factors that prevent patients and healthcare professionals from providing information about prescription medications differ [9]. Therefore, patients with CKD and patients with HD may have different perceptions of medication.

It is difficult to measure the adherence of actual patients. We consider that confirming the status of the leftover medication with each other is one of the easiest ways to review ongoing treatment and adherence. Therefore, we conducted a questionnaire-based survey to elucidate whether differences in perception regarding medications and confirmation of leftover medication among CKD patients, HD patients, pharmacists, and physicians would affect their adherence.

## Materials and methods

Setting and participants

Chronic Kidney Disease and Hemodialysis Patients

We provided a questionnaire to outpatients who received treatment from nephrologists at four facilities in Oita prefecture, Japan. Two facilities (Oita Jinzou Naika Matsuyamaiin Clinic and Matsuoka Medical Clinic) were private hospitals, and others (Oita University Hospital and Oka Hospital) were public hospitals. The Oita University Hospital did not have outpatients undergoing maintenance HDs. Therefore, the HD patients were from the other three hospitals. Participants were informed of the purpose of this survey both verbally and in writing. Those who consented to answer this survey responded in writing or verbally to healthcare professionals.

The inclusion criteria were patients with CKD or HD patients who were regularly visiting a nephrologist for treatment and receiving some form of oral medication. The exclusion criteria were people who did not consent to the study, those who could not manage their medication independently, and patients with dementia who could not understand the questions.

Doctors

We requested nephrologists working in various hospitals in Oita prefecture to respond to the questionnaire. Doctors responded via Google Forms (Google LLC, Mountainview, CA, USA) or in writing.

Pharmacists

We requested pharmacists who worked at the Oita University Hospital to answer the questionnaire. The Oita University Hospital is the only university hospital in Oita prefecture. Therefore, there are many hospitals in Oita prefecture that send patients to this hospital. Pharmacists always check and count prescribed patients' pills when a new patient is admitted. Hence, pharmacists working at the Oita University Hospital might recognize adherence in patients from all hospitals in Oita Prefecture with greater precision than doctors. All pharmacists responded through Google Forms.

Questionnaire content

In this study, we focus only on investigating the gap, that is, the differences in the perception of medications and how to deal with leftover medication between CKD patients, HD patients, doctors, and pharmacists. Therefore, we asked the same questions at the same time to CKD patients, HD patients, physicians, and pharmacists. The questionnaires are presented in Appendix A.

Question 1 included opinions on how to affect the concordance from each of the following perspectives: (1) efficacy of the medication; (2) forms of the medication; (3) frequency of taking medication; (4) medical fee; (5) doctor’s advice; (6) adverse effects; and (7) newer medications. We assessed each response on a five-point Likert scale.

Question 2 for patients was, "How often do you tell your doctor about any leftover medication you may have while on prescribed medication?" The choices are as follows: (a) Never have leftover medication. (b) Always mention leftover medication. (c) Only tell if I have many leftover medications. (d) Never tell the doctor, even if I have any leftover drugs.

For physicians, the question was: "How often do you ask your patients about leftover medication?" The choices are as follows: (a) Always ask. (b) Often ask. (c) Never ask about leftover medication.

For pharmacists, the question was, "How do you think patients disclose their leftover medication in all your experience?" The choices are as follows: a) Always ask the doctor. (b) Depends on the doctor. (c) Never ask about leftover medication.

Ethical issues

The ethics committee of Oita University approved this survey (approval no. 1291). We conducted the survey in accordance with the Declaration of Helsinki and the Clinical Trials Act made by the Ministry of Health, Labor, and Welfare of Japan. Written consent was obtained from all participants. The anonymity of the subjects was ensured.

Statistical analysis

We performed an ordinary regression analysis of what factors were associated with how to deal with leftover medication. The explanatory variables were age, patient states (CKD or HD), drug preference (efficacy of medication, forms of medication, frequency of taking the medication, medical fee, doctor’s advice, adverse effects, and newer medications). A p-value of < .05 was considered statistically significant. In logistic regression analysis, the required sample size is said to be 10 times the number of explanatory variables. In this case, there are seven explanatory variables, and each target explanatory category has four levels (three stages). Therefore, it is considered that 210 or more cases are required. R software (V4.2.3) (R: A Language and Environment for Statistical Computing, R Core Team, R Foundation for Statistical Computing, Vienna, Austria, 2023) was used for analysis in this study.

## Results

In this survey, 383 patients (260 patients with CKD and 123 HD patients), 22 nephrologists, and 28 pharmacists responded. Table 1 shows the characteristics of the patients for age and patient states (CKD or HD).

**Table 1:** Age distribution of responders: CKD patients and HD patients CKD: chronic kidney disease; HD: hemodialysis

Age (in years)	CKD (n=260)	HD (n=123)
20’s	4(2.0%)	0
30’s	18(10%)	0
40’s	16(8.0%)	8(5.0%)
50’s	27(14%)	24(11%)
60’s	58(30%)	27(14%)
70’s	73(40%)	39(27%)
80’s	52(29%)	21(17%)
Over 90’s	12(9.0%)	2(1.6%)

The response rate of valid answers was more than 90% for each category of participants. Table 2 shows the perceptions regarding medication of each group: CKD patients, HD patients, doctors, and pharmacists.

**Table 2:** The percentage of respondents shows who answered "most important" or "important" for each question CKD: chronic kidney disease; HD: hemodialysis

	Patients with CKD	Patients with HD	Doctors	Pharmacists
The efficacy of medications	75.2 %	64.7 %	95.3 %	95.0 %
Forms of medications	45.0 %	46.4 %	66.7 %	90.0 %
Frequency of medications	53.4%	42.5 %	71.4 %	90.0 %
Price of medications	60.5%	47.5 %	19.1 %	90.0 %
Doctor's advise	58.6 %	49.2 %	90.5 %	70.0 %
Side effects of medications	79.6 %	67.8 %	100 %	70.0 %
Newly developed medications	53.7 %	49.2 %	9.5 %	60.0 %

The actual results of the respondents' responses are captured in Appendix B. Tables 3-5 show the answer to the question "How to deal with leftover medication?".

**Table 3:** Responses of CKD patients and HD patients to the question "How to deal with leftover medication?" CKD: chronic kidney disease; HD: hemodialysis

Responses	CKD patients (n=260)	HD patients(n=123)
Never have leftover medication	49 (19 %)	42 (35 %)
Always tell leftover medication	101 (39 %)	11 (9.1 %)
Only tell if I have many leftover medications	89 (35 %)	43 (36 %)
Never tell the doctor even if I have any leftover drug	17 (6.6 %)	25 (21 %)

**Table 4:** Responses of physicians to the question "How often do they confirm leftover medication?"

Responses	Numbers (n=22)
Always	9 (40.9 %)
Often	8 (36.4 %)
Never	5 (22.7 %)

**Table 5:** Responses of pharmacists to the question "How often do you think patients tell their attending physicians about the leftover medication?"

Responses	Numbers (n=28)
Always tell	7 (25.9%)
Depend on doctors	16 (59.3%)
Never tell the doctor even if I have any leftover medication	4 (14.8%)

In particular, 19% of CKD patients and 34% of HD patients informed doctors that they had no leftover medication; 41% of CKD patients and 56% of HD patients never informed the doctor about leftover medication or only informed them that there were many leftover medications. On the other hand, 23% of doctors have never asked their patients about leftover medications. Pharmacists considered the relationship between doctors and patients to be the most important issue in deciding whether the patient informed them about leftover medication or not. Ordinary regression analysis revealed that there is no significant relationship between the state of the patient or the patient’s preferences and how often the patients talk about the leftover medication (Table 6).

**Table 6:** Ordinary logistic regression analysis: what factors were associated with how often you mention leftover medication Ordinary objective values are the way in which patients tend to mention leftover medication (always mention leftover medication, only mention if I have many leftover medications, and never mention even if I have leftover medication). The explanatory variables were age (by 10 years), patients' states (CKD or HD), and preference for medications (five points of the Likert scale: efficacy of the medication, forms of the medication, frequency of taking medication, medical fee, doctor’s advice, adverse effects, and newer medications). Thirty-three responses were omitted because they were incomplete answers. CKD: chronic kidney disease; HD: hemodialysis; CI: confidence interval

Parameter	Odds ratios	p-value	95% CI
Age	0.92	0.26	[-0.22, 0.06]
Patient states (HD for CKD)	1.26	0.30	[-0.21, 0.67]
The efficacy of medications	1.02	0.83	[-0.19, 0.26]
Forms of medications	1.01	0.35	[-0.09, 0.27]
Frequency of medications	1.15	0.16	[-0.05, 0.33]
Price of medications	1.02	0.86	[-0.17, 0.21]
Doctor's advice	0.99	0.89	[-0.22, 0.19]
Side effects of medications	0.99	0.91	[-0.24, 0.22]
Newly developed medications	0.81	0.07	[-0.42, 0.01]

## Discussion

According to the findings of this study, 23% of doctors, 41% of CKD patients, and 56% of HD patients do not seem to discuss leftover medication. Most pharmacists (about 60%) believe that the patient-doctor relationship is an important factor in determining whether a patient informs the doctor about their leftover medication. Indeed, adherence is said to be influenced by the patient-doctor relationship, and therefore the patient-doctor relationship could affect discussing leftover medication with each other [10-14].

Patients with CKD tend to inform their doctor of their excess medication more frequently than HD patients. This may be affected by the health insurance system in Japan. Patients with CKD must pay more money (usually 20%-30% of the entire fee) than hemodialysis patients (they have an upper limit on the fee, from 10,000 to 20,000 yen depending on their income). On the contrary, patients who never had leftover medication were much higher in HD patients compared to CKD patients. One reason is possibly the difference between CKD patients and HD patients: the frequency of hospital visits and awareness of the disease affect the behavior of some HD patients, making them more adherent than CKD patients [15]. The other reason is the patient's background. Hemodialysis patients were older than CKD patients. Older age has been shown to be a positive factor for good adherence [3].

Regarding drug perception, the efficacy and side effects of the medications were regarded as the most important factors. Furthermore, medical fees and the doctor's advice showed the gap between doctors and patients: patients tended to think of more important factors than medical forms and frequency, which was opposite to the doctor's views. This means that healthcare professionals should know and talk about medical costs for patients when prescribing medications.

There are many ways to measure adherence: counting pills, asking patients directly, and measuring the blood for concentrations of drug metabolites [16]. Burnier et al. reported that an electronic system to monitor adherence was the most effective method [17]. However, these systems are expensive and cannot be used by all patients in the real world. Patients usually tend to hide their non-compliant behavior, like leftover medication. Therefore, in our opinion, in the aspect of concordance, it may be better to ask the patients, "Do you have leftover medication?" instead of "Do you take medicine properly?" If a patient informed us about his leftover medication, we could select non-adherent patients effectively, save costs, and take further steps to improve or establish "concordance", talk about shared goals, drug preference, the gap in perception for medications, or something else that disturbs adherence. This attempt might apply to other chronic diseases.

This study has some limitations. First, the doctors who participated in this survey are only from part of the region, Oita prefecture, Japan. Chronic kidney disease patients and hemodialysis patients were in four facilities. The pharmacists were all from one single facility - the Oita University Hospital. The number of doctors and pharmacists was few compared to the patients. The patients in four facilities were treated by certified nephrologists. Although these helped us obtain more than 400 responses, they might cause representative problems and selection bias. Second, we cannot assess the background of the responders and the nonresponses because we cannot obtain these data. Third, the questions used in this study have not been validated because no similar studies have been conducted before. Further studies are needed to clarify the effect of confirming leftover medications on concordance and clinical outcomes.

## Conclusions

Within the limitations of this study, patients and physicians have different perceptions of their medications, and the patient-doctor relationship might affect the leftover medication. Confirming the presence of leftover medication and explaining the reason behind it could be the first and most effective step in decreasing treatment without adherence.

## Figures and Tables

**Figure 1:**
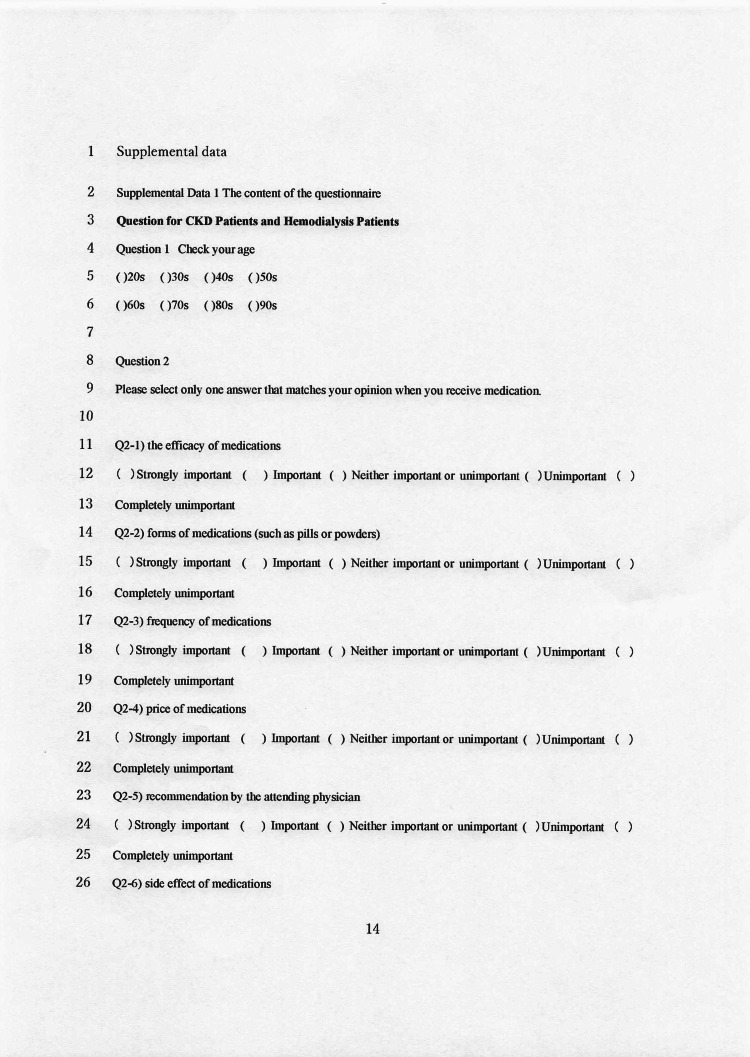
The contents of the questionnaire sent to the respondents, 1/3

**Figure 2:**
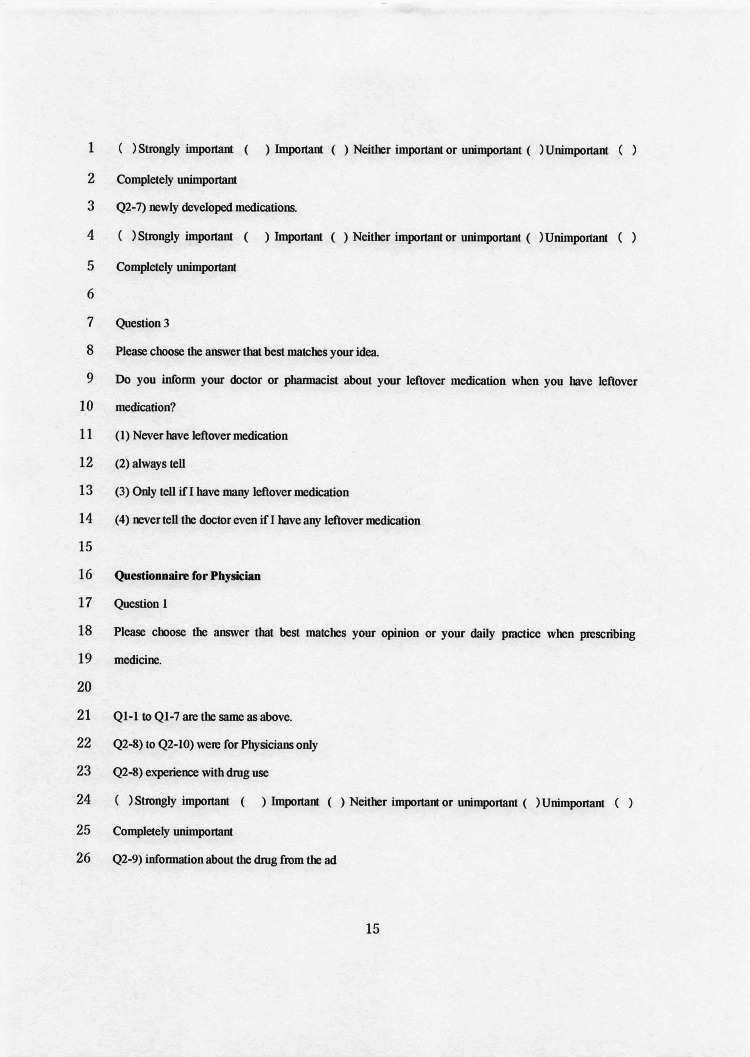
The contents of the questionnaire sent to the respondents, 2/3

**Figure 3:**
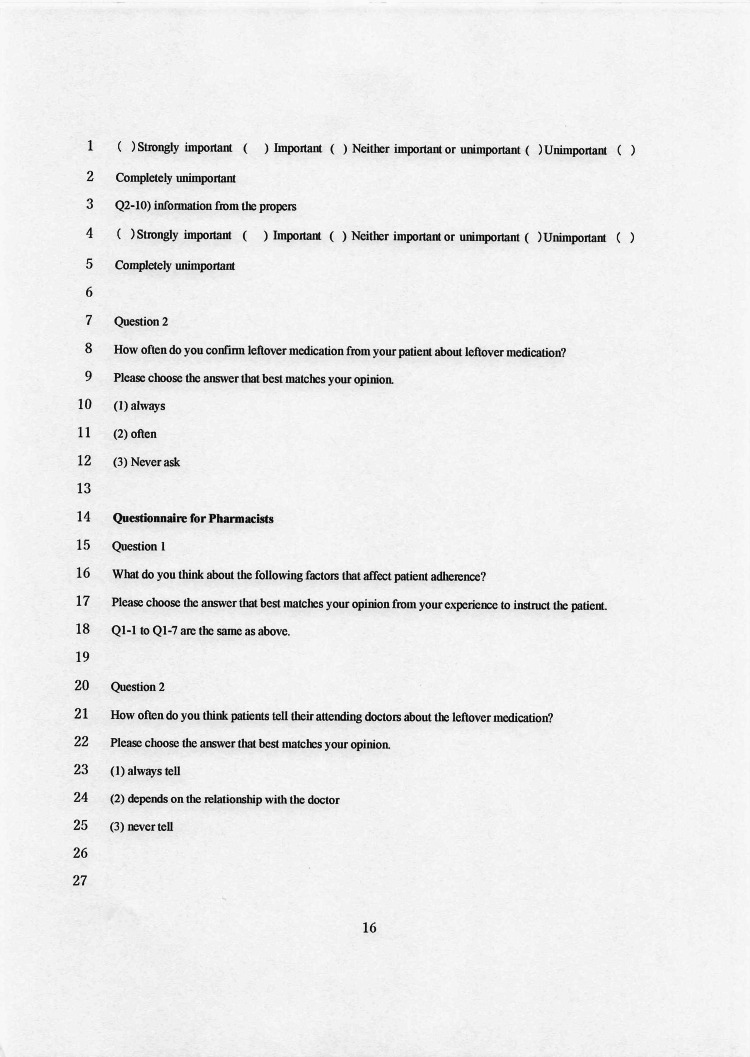
The contents of the questionnaire sent to the respondents, 3/3

## Appendices

Appendix A

The Contents of the Questionnaire

Appendix B 

Responses to Questions by the Respondents

**Table 7:** Responses to questions 2-1 to 2-7 by CKD patients (n=260) CKD: chronic kidney disease

Question	Strongly important	Important	Neither important nor unimportant	Unimportant	Completely unimportant
The efficacy of medications	84 (32.6 %)	110 (42.6 %)	24 (9.3 %)	33 (12.8 %)	7 (2.7 %)
Forms of medications	32 (12.3 %)	85 (32.7 %)	26 (10.0 %)	83 (31.9 %)	34 (13.1 %)
Frequency of medications	43 (17.1 %)	91 (36.3 %)	30 (12.0 %)	71 (38.3 %)	16 (6.4 %)
Price of medications	53 (21.1 %)	99 (39.4 %)	53 (21.1 %)	33 (13.1 %)	13 (5.2 %)
Doctor's advise	69 (27.7 %)	77 (30.9 %)	28 (11.2 %)	57 (22.9 %)	18 (7.2 %)
Side effects of medications	119 (46.7 %)	84 (32.9 %)	14 (5.5 %)	27 (10.6 %)	11 (4.3 %)
Newly developed medications	40 (15.8 %)	96 (37.9 %)	48 (19.0 %)	54 (21.3 %)	15 (5.9 %)

**Table 8:** Responses to questions 2-1 to 2-7 by HD patients (n=123) HD: hemodialysis

Question	Strongly important	Important	Neither important nor unimportant	Unimportant	Completely unimportant
The efficacy of medications	30 (22.4 %)	52 (42.3 %)	13 (10.6 %)	22 (17.9 %)	6 (4.9 %)
Forms of medications	14 (11.4 %)	43 (35.0 %)	16 (13.0 %)	37 (30.1%)	13 (10.6 %)
Frequency of medications	19 (15.8 %)	32 (26.7 %)	16 (13.3 %)	42 (35.0 %)	11 (9.2 %)
Price of medications	14 (11.7 %)	43 (35.8 %)	21 (17.5 %)	33 (27.5 %)	9 (7.5 %)
Doctor's advise	21 (17.5 %)	38 (31.7 %)	22 (18.3 %)	32 (26.7 %)	7 (5.8 %)
Side effects of medications	37 (30.6 %)	45 (37.2 %)	6 (5.0 %)	22 (18.2 %)	11 (9.1 %)
Newly developed medications	11 (9.1 %)	42 (34.7 %)	21 (17.4 %)	35 (28.9 %)	12 (9.9 %)

**Table 9:** Responses to questions 2-1 to 2-7 by pharmacists (n=28)

	Strongly important	Important	Neither important nor unimportant	Unimportant	Completely unimportant
The efficacy of medications	12 (60 %)	7(35 %)	1 (5.0 %)	0 (0.0 %)	0 (0.0 %)
Forms of medications	12 (60 %)	6 (30 %)	0 (0.0 %)	2 (10.0 %)	0 (0.0 %)
Frequency of medications	7 (35 %)	11 (55 %)	2 (10 %)	0 (0.0 %)	0 (0.0 %)
Price of medications	8 (40 %)	10 (50 %)	1 (5.0 %)	1 (5.0 %)	0 (0.0 %)
Patients' demand	8 (40 %)	6 (30 %)	5 (25 %)	1 (5.0 %)	0 (0.0 %)
Side effects of medications	3 (15 %)	11 (55 %)	6 (30 %)	0 (0.0 %)	0 (0.0 %)
Newly developed medications	3 (15 %)	9 (45 %)	5 (25 %)	2 (10 %)	1 (5.0 %)

**Table 10:** Responses to questions 2-1 to 2-10 by doctors (n=22)

	Strongly important	Important	Neither important nor unimportant	Unimportant	Completely unimportant
The efficacy of medications	17 (81 %)	3 (14.3 %)	1 (4.8 %)	0 (0.0 %)	0 (0.0 %)
Forms of medications	3 (14.3 %)	11 (52.4 %)	6 (28.6 %)	1 (4.8 %)	0 (0.0 %)
Frequency of medications	2 (9.5 %)	13 (61.9 %)	3 (14.3 %)	2 (9.5 %)	1 (4.8 %)
Price of medications	1 (4.8 %)	3 (14.3 %)	10 (47.6 %)	6 (28.6 %)	1 (4.8 %)
Patients' demand	5 (23.8 %)	14 (66.7 %)	2 (9.5 %)	0 (0.0 %)	0 (0.0 %)
Side effects of medications	13 (61.9 %)	8 (38.1 %)	0 (0.0 %)	0 (0.0 %)	0 (0.0 %)
Newly developed medications	0 (0.0%)	2 (9.5 %)	12 (57.1 %)	4 (19.0 %)	3 (14.3 %)
Experience	7 (33.3 %)	11 (52.4 %)	2 (9.5 %)	1 (4.8 %)	0 (0.0 %)
Information from advertisements	3 (14.3 %)	11 (52.4 %)	7 (33.3 %)	0 (0.0 %)	0 (0.0 %)
Information from papers	0 (0.0 %)	7 (33.3 %)	10 (47.6 %)	3 (14.3 %)	1 (5.0 %)
